# Alzheimer therapy with an antibody against N-terminal Abeta 4-X and pyroglutamate Abeta 3-X

**DOI:** 10.1038/srep17338

**Published:** 2015-12-02

**Authors:** Gregory Antonios, Henning Borgers, Bernhard C. Richard, Andreas Brauß, Julius Meißner, Sascha Weggen, Vladimir Pena, Thierry Pillot, Sarah L. Davies, Preeti Bakrania, David Matthews, Janet Brownlees, Yvonne Bouter, Thomas A. Bayer

**Affiliations:** 1 Georg-August-University Göttingen, University Medicine Göttingen, Department of Psychiatry and Psychotherapy, Division of Molecular Psychiatry, 37075 Göttingen, Germany; 2 Department of Neuropathology, Heinrich Heine University Düsseldorf, Düsseldorf, Germany; 3 Macromolecular Crystallography, Max-Planck Institute of Biophysical Chemistry (MPI-BPC), 37077 Göttingen, Germany; 4 SynAging, 54000 Nancy, France; 5 Medical Research Council Technology, 1-3 Burtonhole Lane, London, UK, NW7 1AD.

## Abstract

Full-length Aβ1-42 and Aβ1-40, N-truncated pyroglutamate Aβ3-42 and Aβ4-42 are major variants in the Alzheimer brain. Aβ4-42 has not been considered as a therapeutic target yet. We demonstrate that the antibody NT4X and its Fab fragment reacting with both the free N-terminus of Aβ4-x and pyroglutamate Aβ3-X mitigated neuron loss in Tg4-42 mice expressing Aβ4-42 and completely rescued spatial reference memory deficits after passive immunization. NT4X and its Fab fragment also rescued working memory deficits in wild type mice induced by intraventricular injection of Aβ4-42. NT4X reduced pyroglutamate Aβ3-x, Aβx-40 and Thioflavin-S positive plaque load after passive immunization of 5XFAD mice. Aβ1-x and Aβx-42 plaque deposits were unchanged. Importantly, for the first time, we demonstrate that passive immunization using the antibody NT4X is therapeutically beneficial in Alzheimer mouse models showing that N-truncated Aβ starting with position four in addition to pyroglutamate Aβ3-x is a relevant target to fight Alzheimer’s disease.

The discovery that certain early-onset familial forms of Alzheimer disease (AD) appear to be caused by an enhanced precipitation of Aβ peptides led to the hypothesis that amyloidogenic Aβ is intimately involved in the AD pathogenic process^1^. There is evidence that the primary pathology in AD is triggered by oligomeric species and β-sheet containing amyloid fibrils derived from full-length Aβ_1-42_^2^,^3^,^4^,^5^. Numerous variants of Aβ_1-42_ oligomers have been discussed as pathological factors in AD (reviewed in^6^). We have recently pointed out that the biophysical characteristics of Aβ_1-42_ oligomers prefer aggregation into inert amyloid plaques in contrast to N-truncated Aβ_4-42_ and pyroglutamate Aβ_3-42_ (Aβ_pE3-42_) peptides having a higher tendency to stay soluble and maintaining their toxic profile for a longer time period^7^,^8^. Although Aβ_4-42_ is highly abundant in AD brains and was discovered as the first N-truncated peptide^9^ its possible role in AD pathology has been largely overlooked. Among different Aβ species present in AD plaques, Lewis *et al.*^10^ demonstrated that Aβ_4-42_ is an abundant species in AD, aged controls and vascular dementia patients. Using immunoprecipitation in combination with mass spectrometry, Portelius and colleagues^11^ corroborated these findings, reporting that Aβ_4-42_ belong to one of the major species in the hippocampus and cortex of AD patients. N-terminal deletions, including Aβ_4-42_, enhance generation of soluble Aβ oligomers^12^. *In vitro* and *in vivo* exposure indicated that Aβ_4-42_ is as toxic as Aβ_pE3-42_ and Aβ_1-42_. Expression of Aβ_4-42_ in the brain of transgenic mice (Tg4-42 transgenic line) induced a massive CA1 pyramidal neuron loss in the hippocampus without any plaque formation correlating with age-dependent spatial reference memory deficits^13^.

Schenk *et al.*^14^ introduced active immunization as a therapeutic option for AD. APP transgenic mice were immunized with pre-aggregated Aβ_1-42_ preventing the development of plaque formation and astrogliosis. Moreover, vaccination also protected transgenic mice from learning and age-related memory deficits^15^. Many immunization trials in symptomatic AD patients are ongoing and have been extensively reviewed recently^16^.

In the current report, we introduce the monoclonal antibody and the Fab fragment NT4X specific for N-truncated Aβ_4−x_ and Aβ_pE3-42_ as an improved therapeutic entity for AD and study its effect in three mouse models. We also compared the treatment effect of the pan-pyroglutamate Aβ_3−x_ (1–57^17^) antibody, which has a similar plaque-binding profile like two other pyroglutamate Aβ_3−x_ specific antibodies used in preclinical trials^18^,^19^ with the effect of NT4X in 5XFAD mice. The aim of the project was to explore whether Aβ_4−x_ is a relevant drug target in AD.

## Results

### NT4X binds N terminally truncated amyloid peptides

**Figure 1:**
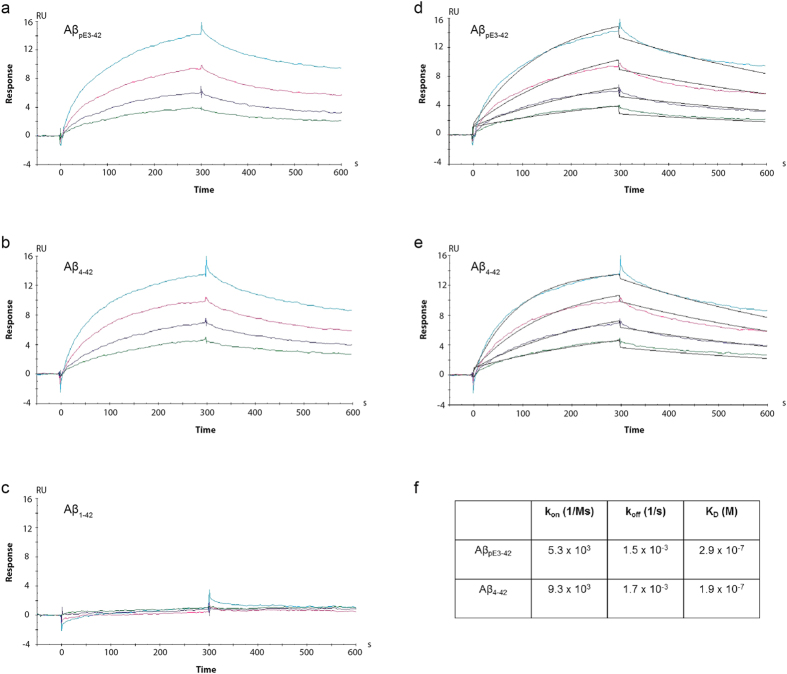
NT4X antibody binds specifically to Aβ_pE3-42_ and Aβ_4-42_ peptides. Sensorgram profiles generated by binding studies using the Biacore T200 showing the association and dissociation binding kinetics of the NT4X antibody to freshly prepared Aβ_pE3-42_ (**a**), Aβ_4-42_ (**b**) and Aβ_1-42_ (**c**) peptides. NT4X binds to Aβ_pE3-42_ and Aβ_4-42_ but does not bind to Aβ_1-42_. The heterogeneous nature of the Aβ peptides limits this application in obtaining accurate kinetic data. However, the theoretical fit of binding of Aβ_pE3-42_ (**d**) and Aβ_4-42_ (**e**) peptides to the NT4X antibody and their kinetics of binding (**f**) is also shown. Abbreviations: RU: response units; K_on_: association rate, K_off_: dissociation rate; KD: equilibrium constant.

We have previously shown that the monoclonal antibody NT4X reacts with N-terminally truncated Aβ_pE3-40/42_ and Aβ_4-40/42_ variants, but not with Aβ_1–40/42_ under denaturing and native conditions^20^ and now confirm this binding profile with direct biophysical binding experiments using Biacore technology (Fig. 1). NT4X binds both Aβ_pE3-42_ and Aβ_4-42_ oligomers but not the full length 1-42 amyloid oligomers. Interestingly, if the peptides are allowed to aggregate over longer time periods, NT4X no longer binds (data not shown).

### NT4X protects primary neurons from N terminally truncated amyloid peptide induced cell toxicity *in vitro*

**Figure 2:**
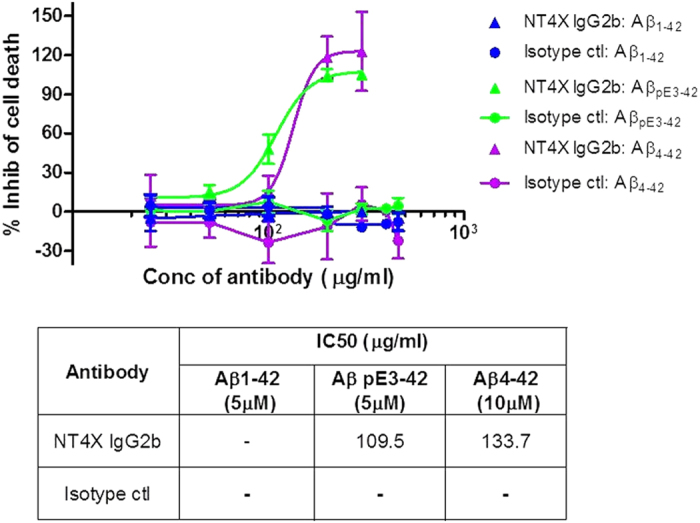
NT4X protection of Aβ_pE3-42_ and Aβ_4-42_ induced toxicity in primary rat neuronal cultures. NT4X IgG2b and an isotype control antibody were assayed to assess potential protective properties in an amyloid peptide induced cellular toxicity assay using primary rat cortical cultures. Cellular toxicity was measured using an LDH release assay and results converted to percentage inhibition of cell death compared to control wells (n = 3 repeats on cell preparations from 3 separate rats). Values plotted are means +/− SEM. The NT4X IgG2b antibody shows inhibition of Aβ_pE3-42_ and Aβ_4-42_ induced toxicity but has no effect on the toxicity induced by full length Aβ_1-42._

*In vitro* assays were set up using primary rat cortical cultures to investigate the protective properties of the NT4X antibody against the various amyloid peptides. Concomitant with the binding properties of the antibody, NT4X rescues Aβ_pE3-42_ and Aβ_4-42_ oligomer induced toxicity but not of full-length Aβ_1-42_ induced toxicity (Fig. 2).

In the current study, we used NT4X and its Fab fragment to elucidate their therapeutic potential in three Alzheimer mouse models.

### Age-dependent spatial reference memory deficits in Tg4-42_hom_ mice

Memory deficits have previously been described in Tg4-42_hom_ mice at 8 months of age^13^. In order to identify the onset of spatial reference memory deficits Tg4-42_hom_ mice were analyzed at four, five, six and seven months (m) of age using the Morris water maze.

**Figure 3:**
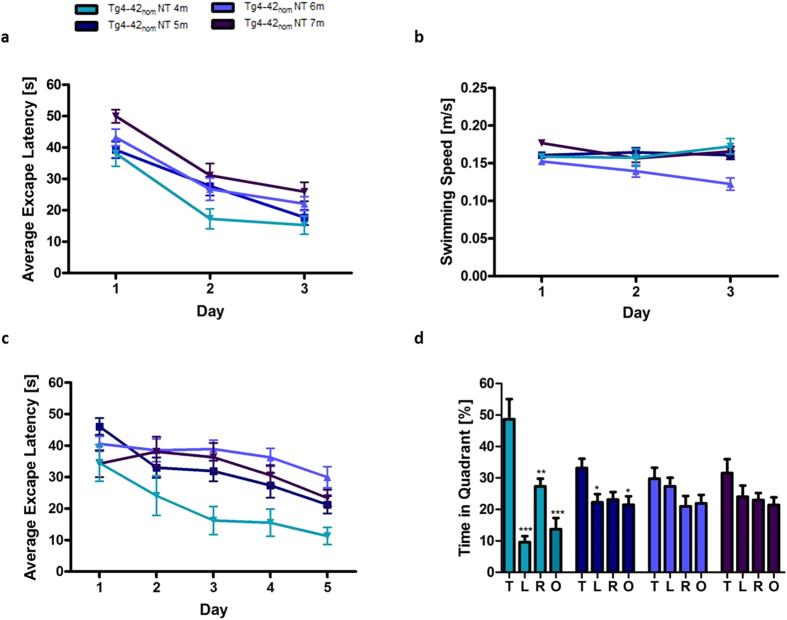
Age-dependent spatial reference memory deficits in homozygous Tg4-42 (Tg4-42_hom_) mice. Spatial reference memory in Tg4-42_hom_ mice was assessed at 4, 5, 6 and 7 months of age using the Morris water maze. (**a,b**) Cued training of the water maze revealed that Tg4-42_hom_ exhibited intact eyesight and the motor abilities to perform the test. (**b**) Tg4-42_hom_ mice showed comparable swimming speeds. (**c**) Spatial learning was assessed using the acquisition training of the Morris water maze. All Tg4-42_hom_ mice showed reduced escape latency over 5 days of acquisition training irrespectively of age. (**d**) A probe trial was given at the end of the learning phase to assess spatial reference memory. Quadrant preference was analyzed for 60 seconds. Tg4-42_hom_ showed no impairment in spatial reference memory at 4 months of age as mice spent a significant greater percentage of the time in the target quadrant. At 5 months of age, spatial reference learning is already slightly impaired. Furthermore, the probe trial revealed a significantly reduced learning behavior for 6- and 7-month-old Tg4-42_hom_ mice as they showed no preference for the target quadrant. Four months old Tg4-42_hom_ mice performed significantly superior to 6-months-old mice on day 3 to 5 (one-way-ANOVA, day 3: p < 0.001; day 4 and 5: p < 0.01). Furthermore, 4-month-old mice showed significantly shorter escape latencies compared to 5- and 7-month-old mice on day 3 (one-way-ANOVA, p < 0.05). ***p < 0.001; **p < 0.01; *p < 0.05; n = 8–17 per group (4 m: n = 8, 5 m: n = 16, 6 m: n = 17, 7 m: n = 9). Swimming Speed and escape latency: repeated measures ANOVA followed by Bonferroni multiple comparisons. Quadrant preference: one-way analysis of variance (ANOVA) followed by Bonferroni multiple comparisons. *T* target quadrant, *L* left quadrant, *R* right quadrant, *O* opposite quadrant. Data presented as mean ± S.E.M; m = months.

All mice showed progressively decreased escape latencies over three days of cued training (Fig. 3a; two-way repeated measures ANOVA, 4 m, 5 m, 6 m and 7 m: main effect of *days*: *p* < 0.0000001). All mice showed comparable swimming speeds (Fig. 3b).

Across the five days of acquisition training Tg4-42_hom_ mice, irrespective of age, showed a significant decrease in the escape latencies (Fig. 3c). For the escape latency a significant main effect of days could be found (two-way repeated measures ANOVA, *days*: *p* = 0.0000001). However, a significant difference was detected between the age groups (two-way repeated measures ANOVA, *age*: *p* = 0.000219). Four months old Tg4-42_hom_ mice performed significantly superior to 6-months-old mice on day 3 to 5 (one-way-ANOVA, day 3: p < 0.001; day 4 and 5: p < 0.01). Furthermore, 4-month-old mice showed significantly shorter escape latencies compared to 5- and 7-month-old mice on day 3 (one-way-ANOVA, p < 0.05).

48 hours after the last acquisition trial, a probe trial was conducted to assess spatial reference memory. At four months of age, Tg4-42_hom_ mice displayed a significant preference for the target quadrant, as indicated by the percentage time spent in the four quadrants of the pool (Fig. 3d, one-way ANOVA, 4m: *p* < 0.001 target vs. left and opposite quadrant, *p* < 0.01 target vs. right quadrant). Five months old Tg4-42_hom_ only spent a significant higher percentage of time in the target quadrant in comparison to the left and the opposite quadrant (one-way ANOVA, 5m: *p* < 0.05 target vs. left and opposite quadrant). Therefore, 5-month-old Tg4-42_hom_ mice showed early signs of impaired spatial reference memory. Moreover six and seven-month-old Tg4-42_hom_ mice displayed no significant preference for the target quadrant indicating severe spatial reference memory deficits.

A significant difference was detected between the age groups (two-way repeated measures ANOVA, age: p = 0.000219). Four months old Tg4-42_hom_ mice performed significantly superior to 6-months-old mice on day 3 to 5 (one-way-ANOVA, day 3: p < 0.001; day 4 and 5: p < 0.01). Furthermore, 4-month-old mice showed significantly shorter escape latencies compared to 5- and 7-month-old mice on day 3 (one-way-ANOVA, p < 0.05).

In summary, the results of the probe trial demonstrated that spatial reference memory deficits in Tg4-42_hom_ mice start at 5 months of age. Beginning at 6 months spatial reference memory in Tg4-42 mice is severely impaired.

### Tg4-42_hom_ mice exhibit age-dependent progressive neuron loss in the CA1 layer of the hippocampus

**Figure 4:**
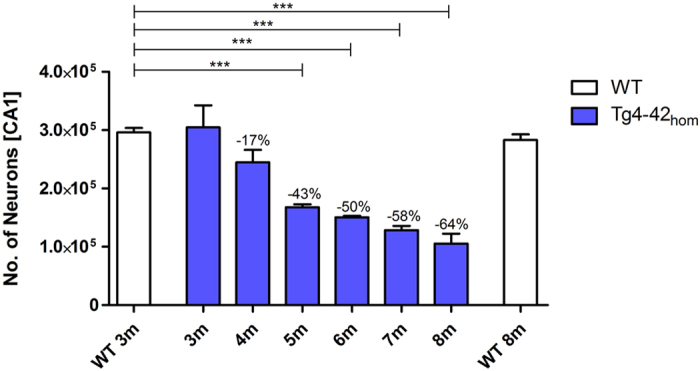
Age-dependent neuron loss in hippocampus of Tg4-42_hom_ mice. Quantification of neurons in the CA1 using unbiased stereology. Tg4-42_hom_ show an age dependent reduction in number of neurons in the CA1 region, which is significant at 5 m, 6 m, 7 m, and 8 m. One-way analysis of variance (ANOVA) followed by Bonferroni multiple comparisons; n = 4–8; ***p < 0.001; data presented as mean ± S.E.M; m = months.

The Tg4-42 transgenic mouse is one of the few transgenic Alzheimer models that exhibit significant neuron loss. Our previous work demonstrated an age- and gene-dose-dependent reduction of neurons in the CA1 layer of the hippocampus in Tg4-42 mice^20^. At eight months stereology revealed a 38% neuron loss in hemizygous Tg4-42 mice compared to wild-type littermate controls. In homozygous Tg4-42 (Tg4-42_hom_) mice the neuron loss was even more pronounced with a more than 60% decline^13^. To extend these findings and determine the progression of neuron loss, CA1 neurons of Tg4-42_hom_ were counted between three and eight months of age using design-based stereology (Fig. 4). Next to Tg4-42_hom_ wild-type mice were analyzed at three and eight months of age.

The hippocampal pyramidal cell layer quantification revealed an age-dependent neuron loss in Tg4-42_hom_ mice (Fig. 4, one-way ANOVA, *age*: p = 0.000001). At three and four months of age no significant difference in the number of neurons was detected between wild-type (*3m*: mean = 296,262; SEM ± 7,532) and Tg4-42_hom_ mice (*3m*: mean = 304,704; SEM ± 37,750; *4m*: mean = 244,781; SEM ± 21320). However, 5-, 6-, 7- and 8-month-old homozygous mice displayed a significant neuron loss of 43%, 50%, 55% and 64%, respectively (one-way ANOVA: *5m*: mean = 167,823; SEM ± 5,106, *p* < 0.001; *6m*: mean = 153,340; SEM ± 2,686, *p* < 0.001; *7m*: mean = 128,531; SEM ± 7,385, *p* < 0.001, *8m*: mean = 105,388; SEM ± 16,960, *p* < 0.001). As expected, the neuron number of wild-type mice did not significantly change during aging (wild-type *8m*: mean = 283,321; SEM ± 9,423).

These results suggest a progressive death of neurons in Tg4-42_hom_ mice that starts at four months and reaches ~64% loss of CA1 neurons by eight months of age. In summary, the results confirm and extend our previous work^13^. Our data indicate that neuron loss in Tg4-42_hom_ occurs even earlier than previously shown and appears to begin when mice are about four months old. As shown above, five month-old Tg4-42_hom_ mice show onset of spatial reference memory deficits with a clear effect at six months of age. Therefore, we started the therapeutic study in Tg4-42_hom_ mice at three months of age a time point before neuron loss or memory deficits were observed.

### Full-length and Fab fragment of NT4X rescues spatial reference memory deficits in Tg4-42_hom_ mice

Severe spatial reference memory deficits in Tg4-42_hom_ were detected at 5 and 6 months of age (Fig. 3). Therefore, 6-month-old Tg4-42_hom_ mice that had received weekly injections with the full-length or Fab of NT4X-antibody, the IgG antibody and PBS for a period of 12 weeks were assessed using the Morris water maze and compared with untreated Tg4-42_hom_ mice.

**Figure 5:**
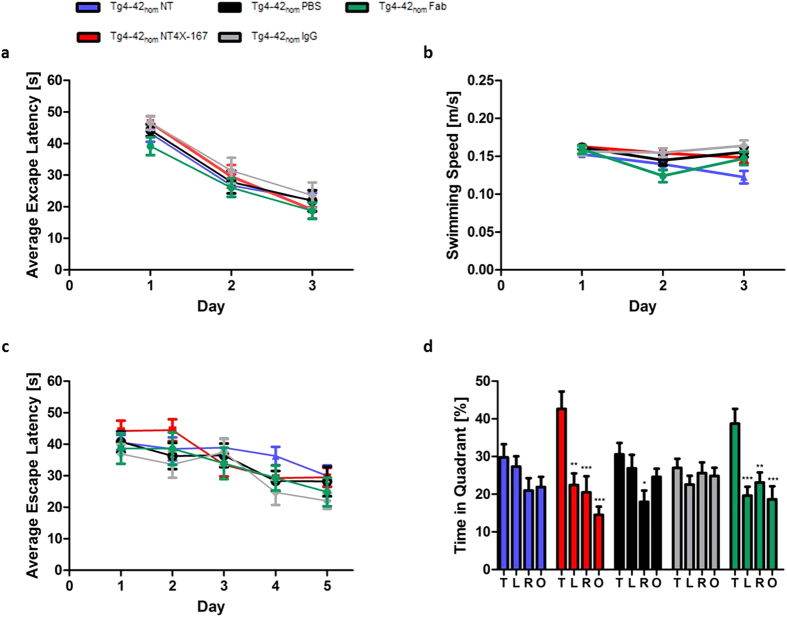
Passive immunization with NT4X rescues learning deficits in Tg4-42_hom_ mice. Tg4-42_hom_ mice that received weekly injections with the full-length NT4X-antibody, the Fab fragment of NT4X, an IgG control antibody and PBS for a period of 12 weeks as well as a group of non-treated (NT) mice were tested at 6 months of age in the Morris Water Maze. (**a,b**) Cued training revealed that all mice exhibited intact eyesight and the motor abilities to perform the test. (**a**) Escape latency decreased progressively over 3 days of cued training for all mice. (**b**) Mice showed comparable swimming speeds. (**c**) Spatial learning was assessed in the acquisition training. All Tg4-42_hom_ mice showed reduced escape latency over the 5 days of acquisition training. (**d**) Spatial reference memory was impaired in non-treated Tg4-42_hom_ as well as in PBS- and IgG-treated Tg4-42_hom_ mice as they showed no preference for the target quadrant in the probe trial. In contrast, Tg4-42_hom_ mice immunized with the full-length NT4X and the Fab fragment of NT4X antibody displayed no learning deficits at this age. ***p < 0.001; **p < 0.01; *p < 0.05. n = 10–17 per group (Tg4-42_hom_ NT: n = 17, Tg4-42_hom_ NT4X: n = 15, Tg4-42_hom_ Fab: n = 10, Tg4-42_hom_ IgG: n = 14, Tg4-42_hom_ PBS n = 15). Swimming Speed and escape latency: repeated measures ANOVA followed by Bonferroni multiple comparisons. Quadrant preference: One-way analysis of variance (ANOVA) followed by Bonferroni multiple comparisons. *T* target quadrant, *L* left quadrant, *R* right quadrant, *O* opposite quadrant. Data presented as mean ± S.E.M; m = months.

Non-treated and treated Tg4-42_hom_ groups showed progressively decreased escape latencies in the cued training (two-way repeated measures ANOVA, *days*: *p* = 0.0000001; Fig. 5a). All mice showed comparable swimming speeds (Fig. 5b). However, non-treated Tg4-42_hom_ swam significantly slower than PBS- and IgG-injected mice on the third day of cued training (one-way ANOVA, PBS *p* < 0.05, IgG *p* < 0.01). The cued training revealed that all mice had an intact vision and appropriate motor abilities to swim.

All groups showed a significant decrease in the escape latencies over the five days of the acquisition training (two-way repeated measures ANOVA, *days*: *p* = 0.0000001, Fig. 5c).

No quadrant preference was found for non-treated, IgG- and PBS-injected Tg4-42_hom_ in the probe trial. Both full-length and Fab NT4X immunized Tg4-42_hom_ mice displayed a significant preference for the target quadrant, as indicated by the percentage time spent in different quadrants of the pool (Fig. 5d, one-way ANOVA, full-length NT4X: *p* < 0.001 target vs. right and opposite quadrant, *p* < 0.01 target vs. left quadrant; Fab NT4X: *p* < 0.001 target vs. left and opposite quadrant, *p* < 0.01 target vs. right quadrant). Therefore, both the full-length NT4X antibody and the Fab fragment of NT4X were able to rescue spatial reference memory deficits in 6-months-old Tg4-42_hom_ mice.

### Full-length and Fab of NT4X decelerate neuron loss in Tg4-42_hom_ mice

**Figure 6:**
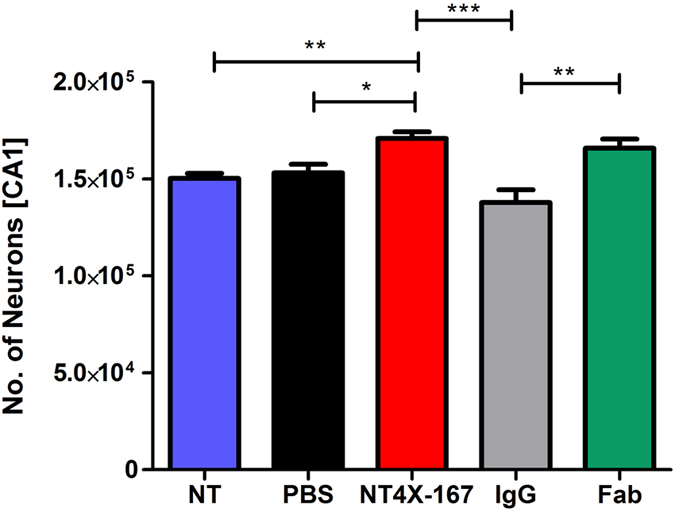
NT4X mitigates neuron loss in Tg4-42_hom_ mice. Quantification of neurons in the CA1 of 6-months-old Tg4-42_hom_ using unbiased stereology. Tg4-42_hom_ mice that received weekly injections with the NT4X antibody or its respective Fab fragment, both an IgG control and a PBS control group for a period of 12 weeks as well as a group of non-treated mice were analyzed at 6 months of age. Tg4-42_hom_ mice immunized with NT4X full length or Fab fragment both displayed significantly more neurons than same-aged untreated, IgG and PBS injected mice. In contrast, the number of neurons did not differ significantly between untreated, IgG and PBS injected Tg4-42_hom_ mice. One-way analysis of variance (ANOVA) followed by Bonferroni multiple comparisons; n = 9–15 (Tg4-42_hom_ NT: n = 9, Tg4-42_hom_ NT4X: n = 15, Tg4-42_hom_ IgG: n = 9, Tg4-42_hom_ PBS n = 15). *p < 0.05; **p < 0.01; ***p < 0.001; data presented as mean ± S.E.M; NT = non-treated.

In order to study the potential therapeutic effect of NT4X on the observed neuron loss in 6 months old Tg4-42_hom_ the neuron numbers in the CA1 were quantified using design-based stereology. The number of neurons was compared between 6-months-old Tg4-42_hom_ immunized with full-length NT4X or Fab NT4X and three same-aged control groups: untreated Tg4-42_hom_ mice; PBS injected Tg4-42_hom_ mice and mice injected with an IgG2b antibody as an isotype control. Stereology revealed a significant treatment effect (Fig. 6, one-way ANOVA, *treatment*: p = 0.000016). Following ANOVA, the individual groups were then analyzed using Bonferroni multiple comparisons. Compared to untreated, PBS injected and IgG injected mice, the neuron numbers in Tg4-42_hom_ were significantly increased after immunization with NT4X (Fig. 6; mean = 171,000; one-way ANOVA: SEM ± 3,414; NT4X vs NT: p < 0.01; NT4X vs PBS: p < 0.05; NT4X vs IgG: p < 0.001). In contrast, the number of neurons did not differ significantly between untreated (mean = 150,300; SEM ± 2,572), PBS injected (mean = 153,100; SEM ± 4,500) and IgG injected Tg4-42_hom_ mice (mean = 137,900; SEM ±6,555). NT4X immunized Tg4-42_hom_ mice displayed 13% more neurons in the CA1 layer of the hippocampus than non-treated mice. Furthermore, Fab injected mice (mean = 165,800; SEM ± 4,717) displayed significantly more neurons than IgG injected control mice. The neuron loss in 6-months-old full-length NT4X immunized mice was comparable to 4.5-months-old Tg4-42_hom_ mice, whereas the neuron loss in Fab-treated mice was comparable to 5-months-old untreated Tg4-42_hom_ mice.

### Full-length and Fab NT4X rescue Aβ4-42 induced working memory deficits in wildtype mice

**Figure 7:**
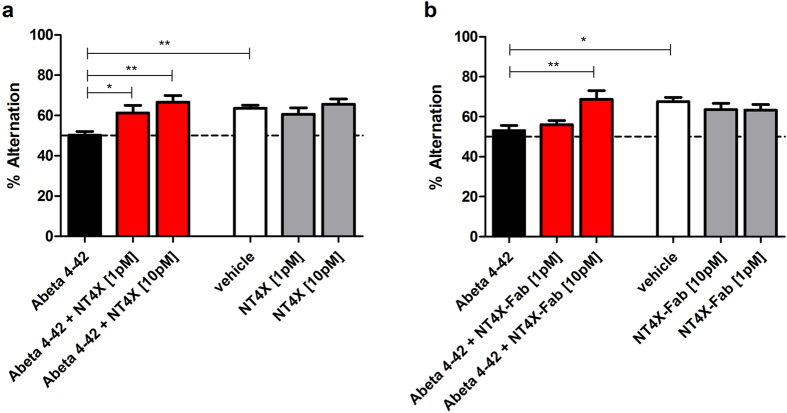
Full-length and Fab fragment of NT4X rescue Aβ_4-42_ induced working memory deficits in wildtype mice. (**A,B**) Working memory deficits were induced by intraventricular injection of Aβ_4-42_ as the mice performing at chance level (dashed line). Both treatment with (**a**) full-length antibody and (**b**) Fab fragment rescued memory deficits in a dose-dependent manner. Mice treated with the vehicle control and the vehicle in combination with full-length or Fab fragment of NT4X respectively demonstrated normal working memory performance. One-way analysis of variance (ANOVA) followed by Bonferroni multiple comparisons; n = 6–8 per group; **p < 0.01; *p < 0.05; data presented as mean ± S.E.M.

In order to study the therapeutic effects of NT4X and its Fab fragment in wild-type mice, the animals were intraventricularly injected with freshly prepared 50 pmol Aβ_4-42_ and Aβ_4-42_ in combination with the NT4X antibody or the Fab fragment, respectively. Working memory was assessed using the Y-maze (Fig. 7). The alternation rate was significantly reduced after injection of Aβ_4-42_ reaching chance level (Fig. 7a,b, dashed line). In contrast, mice injected with Aβ_4-42_ and 1 pmol NT4X antibody behaved like vehicle controls and learned well (compared to Aβ_4-42_ injection; one-way ANOVA: p < 0.05). The alternation rate in mice treated with 10 pmol NT4X further increased (compared to Aβ_4-42_ injection; one-way ANOVA: p < 0.01). Furthermore, mice treated with Aβ_4-42_ and 10 pmol Fab NT4X also showed no deficits in working memory and behaved like the vehicle control (compared to Aβ_4-42_ injection; one-way ANOVA: p < 0.01). Mice treated with only the NT4X antibody or its Fab fragment showed a similar alternation rate than mice treated with the vehicle control alone. Therefore, the NT4X antibody and its Fab fragment were able to rescue the learning deficits induced through Aβ_4-42_ injection in a dose-dependent manner.

### NT4X decreases plaque load in 5XFAD mice

**Figure 8:**
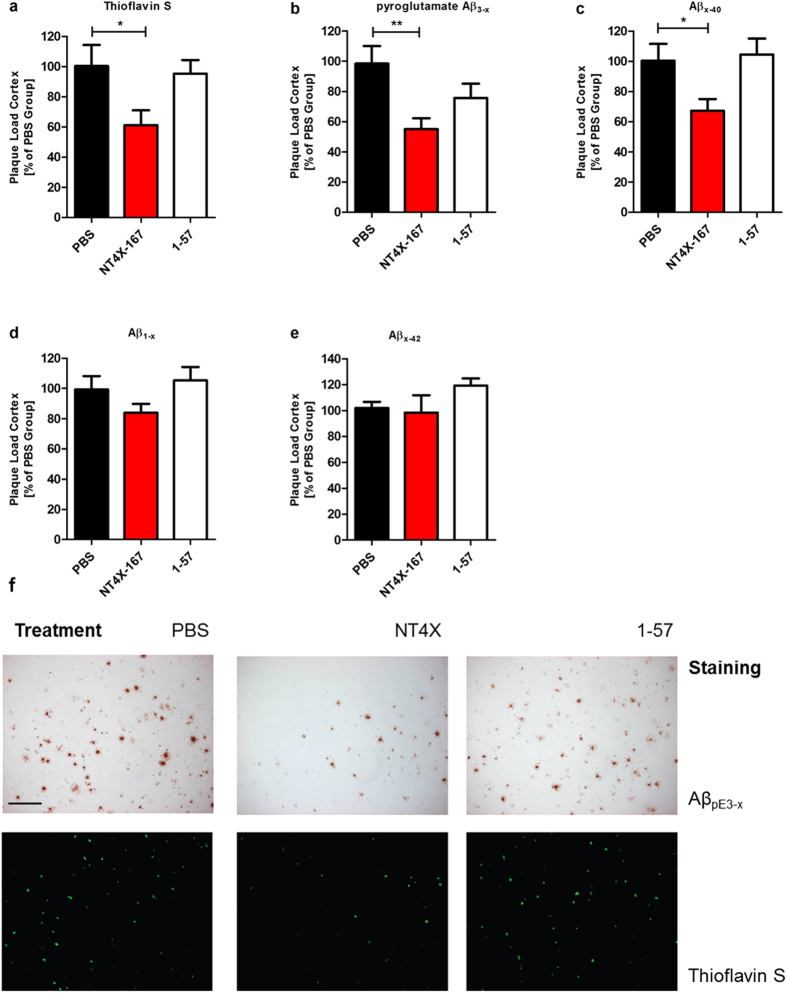
Reduced plaque loads in immunized 5XFAD mice. Plaque load analysis of NT4X and 1–57 immunized 5XFAD mice compared to PBS injected 5XFAD mice. (**a**) NT4X treatment significantly reduced the percentage of brain area occupied by fibrillar Aβ deposits in the cortex in 5XFAD mice demonstrated by Thioflavin S staining. (**b**) Immunostaining with the antibody 1–57 against pyroglutamate Aβ_3−x_ revealed a reduced plaque burden in the cortex of NT4X immunized 5XFAD. D Moreover, immunostaining with an antibody against Aβ_x-40_ showed a reduced plaque burden in the cortex of NT4X immunized 5XFAD mice. (**c,e**) Immunostaining with antibodies againt Aβ_1−X_ and Aβ_X−42_ revealed no significant differences in the plaque load of NT4X or 1–57 immunized 5XFAD and the PBS control group. (**f**) Representative images from Thioflavin S and pyroglutamate Aβ_3-X_ staining for respective treatment groups of PBS, NT4X and 1–57, both showing a decreased staining for NT4X treatment group. One-way analysis of variance (ANOVA) followed by Dunnett’s multiple comparison test against the PBS control group; n = 7–9; **p < 0.01, *p < 0.05 data presented as mean ± S.E.M.

Plaque pathology in 5XFAD mice starts between two and three months of age and increases massively with age^21^. In order to determine the effects of NT4X immunization on the Aβ deposition in 5XFAD mice, brain sections were stained with Thioflavin S (ThS), which selectively detects fibrillar Aβ deposits^22^. NT4X immunized mice showed significantly fewer ThS-positive fibrillar plaques in the cortex as compared to the PBS control group (Fig. 8a, one-way ANOVA, p < 0.05). To further characterize the nature of the deposited Aβ plaques, brain sections were immunostained with N- and C-terminus specific Aβ antibodies. The pan-pyroglutamate Aβ_3-x_ antibody 1–57 was used to explore a possible treatment effect targeting pyroglutamate Aβ_3-x._ 1–57 immunized 5XFAD mice showed no reduced ThS positive plaque load in the cortex. NT4X immunized 5XFAD mice showed significantly fewer pyroglutamate Aβ plaques (Fig. 8b, one-way ANOVA *p* < 0.01) than the PBS control group. In contrast, 1–57 immunized 5XFAD mice showed no reduced plaque load in the cortex. Immunostaining with an anti-Aβ_x-40_ antibody showed a reduced plaque burden in the cortex of NT4X immunized 5XFAD mice (Fig. 8c, one-way ANOVA, p < 0.05). Immunostaining with an anti-Aβ_1-X_ (Fig. 8d) and anti-Aβ_x-42_ (Fig. 8e) antibody revealed no significant differences in the plaque load of NT4X or 1–57 immunized 5XFAD mice and the PBS control group. Thus, NT4X administration decreased fibrillar and total Aβ depositions, especially N-truncated pyroglutamate Aβ_3-x_ in the cortex of 5XFAD mice. Representative images of ThS and pyroglutamate Aβ_3-x_ plaque load is shown in Fig. 8f.

Soluble levels of Aβ_1-40,_ Aβ_1-42_ and Aβ_pE3-42_ were analysed by commercially available ELISAs in total brain extracts of 5XFAD mice (Table 1). Although the levels in the NT4X treated mice was lower for Aβ_1-42_ and Aβ_pE3-42_ the effect was not significant.

## Discussion

While it is known that N-truncated Aβ peptides are major constituents in the brain of Alzheimer patients^8^ the major focus was on the role of pyroglutamate Aβ_3-42_. The possibility of N-truncated Aβ_4-42_ as a drug target has been largely neglected. We have generated a monoclonal antibody, which binds to both Aβ_pE3-42_ and Aβ_4-42_ truncated N-terminal amyloid low molecular weight oligomers but not to full-length Aβ_1-42_ and will protect primary neuronal cultures from toxicity induced by the N-truncated amyloid peptides but not from the full length amyloid peptide.

### Expression of Aβ4-42 induces an age-dependent CA1 neuron loss and associated memory decline

Tg4-42 mice develop severe hippocampus neuron loss and spatial reference memory deficits^13^. The Tg4-42 model represents the first mouse model expressing exclusively N-truncated Aβ_4-42_. In the present study we examined the exact onset of spatial reference memory loss and the associated number of degenerated neurons in homozygous Tg4-42 mice. Broadbent *et al.*^23^ examined the relationship between hippocampal lesion size and spatial memory in rats. Spatial memory impairment started after bilateral dorsal hippocampal lesions that encompassed 30–50% total volume, and as lesion size increased from 50% to 100% of total hippocampal volume, performance was similarly impaired. Moser *et al.*^24^ claimed that only 20–40% of the total hippocampus is required for efficient spatial learning. These findings show that the hippocampus is important for spatial reference memory albeit a significant neuron loss can be compensated. Our findings are in good agreement with these observations. At five months of age, Tg4-42 mice displayed a 43% neuron loss and first but mild signs of spatial reference memory deficits. However, at six months of age, a significant loss in spatial reference memory performance was observed with a reduction of 50% of neurons. We therefore conclude that a loss of 50% CA1 neurons is critical for learning and memory performance.

### Full-length and Fab NT4X mitigated neuron loss and rescued memory decline in Tg4-42 mice

The neuron loss in Tg4-42 mice starts very early with 17% loss at four months of age. This is an attractive time point for therapeutic intervention as the mice are still pre-symptomatic, capable of performing well in the Morris water maze task up to an age of five months. We therefore started the passive immunization treatment at three months for a period of 12 weeks. Full-length NT4X immunized Tg4-42 mice displayed significantly more neurons as compared to control groups, representing a gain of two month with concomitant rescue of functional spatial reference memory in the life of a 6 months old mouse.

### Full-length and Fab NT4X rescued memory after acute exposure to Aβ4-42 oligomers

We have previously shown that intraventricular injection of Aβ_4-42_ oligomers induced deficits in working memory in wild type mice^13^. In the present study, treatment with NT4X and its Fab fragment rescued the deficits in a dosage-dependent manner arguing for a direct neutralizing effect of the antibody to Aβ_4-42_ oligomer toxicity. The use of ICV injected Aβ_4-42_ oligomers is likely better reflecting the conditions found in AD brains in the vicinity of plaques strengthening the relevance of the potential therapeutic use of NT4X.

### NT4X lowers plaque load in 5XFAD mice

Using mass spectrometric analysis, we could previously demonstrate that 5XFAD mice harboured the following Aβ peptides: Aβ_1-42_, Aβ_4-42_, Aβ_5-42_, Aβ_pE3-42_ and Aβ_3-42_^25^. In the present study we treated 5XFAD mice between five and 7.5 months of age. Passive immunization with NT4X lowered plaque load for distinct Aβ species. As NT4X reacts with both Aβ_4-x_ and Aβ_pE3-x_^20^ a decrease of Aβ_pE3-x_ positive plaques can be explained by direct binding to Aβ_pE3-x_ peptides. As an isotype control we used the IgG2b antibody 1–57, which detects plaque-born pan-Aβ_pE3-x_^17^. As treating 5XFAD mice with 1–57 had no effect on Aβ_pE3-x_ levels, we speculate that this pan-pyroglutamate Aβ-specific antibody was absorbed by binding to existing plaques in the 5XFAD mice and was thereby neutralized. Alternatively, it could also be that NT4X lowered Aβ_pE3-x_ levels indirectly if Aβ_4-x_ would act as a seeding factor for Aβ_pE3-x_ peptides. In agreement with this explanation, we previously observed that intraneuronal Aβ_4-x_ preceded the appearance of Aβ_pE3-x_ in 5XFAD mice^20^. Thioflavin-S-positive plaques and Aβ_x-40_ plaques were also significantly reduced after NT4X treatment suggesting a treatment effect against soluble Aβ_x-40_ aggregates either by direct binding of the antibody to the peptides or indirectly by influencing seeding Aβ species like Aβ_pE3-x_ as shown previously^25^,^26^. NT4X does not bind Aβ_1-42_ aggregates^20^. Hence it can be explained why Aβ_1-x_- and Aβ_x-42_-positive plaque load was unchanged.

The treatment effects were only modest. One explanation could be that treatment was started after substantial plaque development. In addition, we would like to point out more forcefully that the “classic” AD mouse models are likely less than ideal models to study the pathogenic role of Aβ_4-x_. Aβ_4-x_ is much less abundant in AD mouse models compared to AD patients (compare Portelius *et al.*^11^ and Wittnam *et al.*^25^, with mass spectrometry performed by the same group). The low abundance of Aβ_4-x_ in 5XFAD mice could be the simplest explanation why anti-Aβ_4-x_ immunotherapy might not yield spectacular effects in models such as 5XFAD. In addition, the 5XFAD model represents a typical model for familial AD producing primarily full-length Aβ_1-42_^21^,^25^, a situation not found in the brain of patients with sporadic AD^8^.

### Mechanism of NT4X treatment effect and advantage of NT4X therapy

While it is evident that active or passive immunotherapy is effective in Alzheimer mouse models the mechanism of action remains elusive. Several hypothesis are currently being discussed^8^. Antibodies may act catalytically to dissolve preformed Aβ aggregates or prevent Aβ aggregation in the CNS^27^. Microglia clearance of Aβ has also being considered^28^. Intracranial administration of anti-Aβ antibodies into Tg2576 transgenic APP mice resulted in clearance of amyloid deposits and was associated with microglial activation^29^. The Fc receptor of microglia may interact with the Fc part of the antibody bound to Aβ^30^. On the contrary, using Fc receptor-gamma chain knock-out mice did not rescue plaque load in APP Tg2576 transgenic mice after passive immunization. Therefore, the Aβ-antibody treatment was not dependent on Fc receptor-mediated phagocytic^31^. Another therapeutic option does not even require the penetration of the blood brain barrier leading to the formulation of the peripheral sink hypothesis^32^. Chronic treatment with the monoclonal anti-Aβ antibody m266 led to increased plasma levels of Aβ and reduced amyloid plaques in the PDAPP transgenic mouse model. This hypothesis has been challenged by another study though. Yamada *et al.*^33^ have reported that immunotherapy with m266 neutralizes intracerebral, rather than peripheral, soluble, monomeric forms of Aβ.

Soluble Aβ and plaque-bound levels do not necessarily correlate with therapeutic outcome. In a preclinical passive immunization study it has been possible to show reversal of memory deficits without reducing brain Aβ burden in an AD model^34^. Passive immunization of APPswe/PS1ΔE9 transgenic mice with an antibody against the N-terminus of pyroglutamate Aβ_3-X_ significantly reduced total plaque deposition in hippocampus and cerebellum, however, Aβ levels measured by ELISA were not affected^35^. De Mattos and colleagues^19^ reported that Aβ antibodies can have different mode of actions in clearing plaque load depending in their target engagement.

The antibody pool reaching the brain could have been neutralized by binding to amyloid plaques, which would lead to weakened efficiacy. This appeared to be the case when we injected antibody 1–57 in 5XFAD mice. This pan-pyroglutamate Aβ antibody detects abundant plaques in 5XFAD mice^17^. Furthermore, the use of antibodies targeting mainly plaques is now being regarded as a potential risk factor as plaques may serve as reservoirs of toxic Aβ peptides^4^,^6^. The failure of Bapineuzumab in clinical trials^36^ could at least be partly explained by the mobilization of toxic Aβ species from inert plaque material. Moreover, the crystal structure of a Bapineuzumab Fab-Aβ peptide complex revealed that it captured Aβ in a monomeric helical conformation at the N-terminus^37^. The authors concluded that the crystal structure explains the antibody’s selectivity for monomeric Aβ species and that it cannot recognize N-terminally modified or truncated Aβ peptides. We showed that both full-length and the Fab fragment of NT4X rescued memory deficits in two mouse models for AD and mitigated neuron loss in the Tg4-42 model. As the Fab fragment lacks the Fc part of the antibody, we conclude that microglial FcR is not involved in neutralizing Aβ_4-42_ and pyroglutamate Aβ_3-42_. Overall, this report is the first to demonstrate the therapeutic potential of NT4X, an antibody recognizing two N-terminally truncated peptides Aβ_4-x_ and pyroglutamate Aβ_3-x_ in three mouse models for AD.

## Material and Methods

### Transgenic mice

The transgenic mouse lines Tg4-42 and 5XFAD used in this study have been described previously^13^,^21^.

### Cell culture and antibody purification

The NT4X hybridoma cell line (IgG2b; official name of cell line Aβ4–40 NT4X-167; DSM ACC3162) was cultured in a serum-free media for hybridoma culture ISF-1 (Biochrom) and maintained in fed batch/continuous perfusion mode at 25% Oxygen, 5% CO2 and 37 °C in a 3L glass vessel bioreactor (Applikon) controlled using ADI 1030 (Applikon). The cell-culture supernatants were harvested by centrifugation at 500 g and the media was collected. Media was further centrifuged at 10,000 g for 30 min prior to Protein A affinity chromatography and pH was stabilized by addition of 20% (v/v) of PBS buffer. Media was loaded at 5 ml/min onto HiTrap™ Protein A HP (GE Healthcare) connected to an AKTAxpress (GE Healtcare). Further column washing and elution was done according to Protein A column manufacturer instructions. The eluted sample was further purified by size-exclusion chromatography using a HiLoad 26/60 Superdex 200 pg run in PBS on an AKTAxpress (GE Healthcare). Fab fragments were produced utilizing papain digestion of the intact NT4X monoclonal antibody. Papain agarose (Sigma) was pre-activated, shaking at 1400 rpm, with 10 mM cysteine (Sigma) in PBS for 30 min at room temperature. Previously purified NT4X antibody (in PBS) was incubated for 4 hours at 37 °C with pre-activated papain agarose (1 U agarose/5 mg antibody). Papain agarose is subsequently removed by filtration through a 0.45 μm filter. Fab sample is further purified by size-exclusion chromatography using a HiLoad 26/60 Superdex 75 pg run in PBS on an AKTAxpress (GE Healthcare).

### Biophysical binding studies: kinetic analysis of Aβ peptides binding to immobilised NT4X antibody

Surface Plasmon Resonance (SPR) analysis was performed at 25 °C using the Biacore T200 (GE Healthcare). Purified NT4X monoclonal antibody (0.2 ug/ml) was captured on a CM5 chip (to which an anti-mouse IgG antibody was immobilised) at a density of ~130 response units (RU) to study the binding kinetics with Aβ_1-42_, Aβ_pE3-42_ and Aβ_4-42_. A plain CM5 surface, which was activated and blocked by ethanolamine was used as a reference surface. Binding experiments were performed using HBS-EP buffer (0.01 M HEPES, pH 7.4, 0.15 M NaCl, 3mM EDTA, 0.005% P20) as the running buffer. The binding sensorgrams were recorded by injecting different concentrations (125nM, 250nM, 500nM and 1000nM) of freshly prepared Aβ peptides in HBS-EP buffer for 5 min at a flow rate of 30μl/min over the immobilised NT4X antibody surface. The dissociation profile was monitored for 20 min, and then the surface was regenerated with 10 mM glycine HCl at pH 1.7. To avoid any mass transfer effect, the experiments were performed at a high flow rate of 30 μl/min using freshly prepared Aβ peptides. Reference subtracted SPR data were evaluated with the Biacore T200 Evaluation Software (version 2.0) using a 1:1 Langmuir binding model.

### Cell based neuroprotection assays

Neurones were prepared from the cortex of 18 day old embryonic CD rat brains using a papain digestion kit (Worthington, Biochemical Corporation). Cells were plated at 3 × 10^5^ cells/ml in 96 well Cell Coat, Poly-D-Lysine coated plates (Greiner) in Neurobasal media with Pen/Strep, L-glutamine and B27 supplement with antioxidants (Gibco, ThermoFisher). The plates were incubated in a humidified incubator at 37^o^C, 5% CO_2_ for two days prior to adding the antibodies and amyloid peptides. Media was removed from the plates and fresh Neurobasal media containing B27 supplement without antioxidants (Invitrogen) added to perform the assay. Purified antibody stocks (mouse NT4X IgG2b and an isotype control antibody) were diluted in sterile PBS (Ca/Mg free) and 10ul added to a total volume of 100 μl per well to give a series of dilutions at the required concentration. HFIP treated and dried amyloid peptide aliquots (stored in a desiccator at −80 °C) were dissolved in 100 mM NaOH and further diluted in Neurobasal media without antioxidants to achieve final peptide concentrations of 5 μM for both Aβ_pE3-42_ and Aβ_1-42_ and 10 μM for Aβ_4-42_ peptide in the cell assay (Anaspec, Fremont, CA (Eurogentec) and California Peptide Research, San Francisco). The plates were incubated for seven days prior to LDH measurement with the CytoTox96 Non-Radioactive cytotoxicity assay kit (Promega). The LDH assay was performed on 50 μl/well culture supernatant in fresh 96 well assay plates (Costar). The plates were read at 490 nm on a Tecan Safire II plate reader. The inhibition assay was repeated n = 2 to 3 for each peptide with the two antibodies detailed.

### Passive immunization against Aβ

The potential therapeutic effects of NT4X were studied using a passive immunization approach in Tg4-42_hom_ and 5XFAD mice.

Passive immunization was performed by intraperitoneal injections of NT4X or its Fab fragment^20^ and compared to control groups using antibody of the same immunoglobulin class as NT4X (IgG2b) and PBS injections. Tg4-42_hom_ mice were immunized by injections of the NT4X antibody, 10 mg/kg body weight, diluted in sterile PBS (pH 7.4). Mice received weekly injections beginning at 3 months of age. Each mouse received a total of 12 injections. Behavior testing started between the ninth and the tenth injection. Animals were sacrificed after the last injection. Control groups comprised of Tg4-42_hom_ mice received intraperitoneal PBS injections or injections of IgG2b antibody (10 mg/kg body weight) diluted in PBS, respectively. Control mice were treated exactly the same as the NT4X group receiving 12 injection of the respective control solution starting at 3 months of age. A non-treated Tg4-42_hom_ group served as an additional control. Animals were sacrificed after the last injection at 6 months of age.

In addition, 5-month-old 5XFAD mice were weekly injected with NT4X (100 μl/10 g body weight, diluted in sterile PBS), 1–57 (IgG2b backbone^17^ against the N-terminus of pyroglutamate Aβ_3−x_; 10 mg/kg body weight, diluted in sterile PBS) or PBS respectively. Each mouse received a total of 10 intraperitoneal injections. Behavior testing started between the 9^th^ and the 10^th^ injection. Animals were sacrificed after the last injection at 7.5 months of age.

### Spatial reference memory by Morris water maze

Spatial reference memory in Tg4-42_hom_ mice was evaluated using the Morris water maze^38^ as described previously^13^.

### Quantification of neuron numbers using unbiased stereology

Stereological analysis was performed as previously described^13^,^39^. The hippocampal cell layer CA1 (Bregma −1.22 to −3.52 mm) was delineated on cresyl violet-stained sections and analysed with a stereology workstation (Olympus BX51 with a motorized specimen stage for automatic sampling), StereoInvestigator 7 (MicroBrightField, Williston, USA) and a 100x oil lens (NA = 1.35).

### Immunohistochemistry and histology

Mouse tissue samples were processed as described previously^17^. Aside from 1–57 (pyroglutamate Aβ_3-x_, 1 mg/ml; 1:5000) the following antibodies were used: mouse monoclonal G2-10 (Aβ_X-40_, Millipore, Schwalbach), rabbit polyclonal antibodies against Aβ_42_ (Aβ_X-42_, #218703, Synaptic Systems, Göttingen) and 82E10 (Aβ_1-x_, 82E1; 1 μg/ml, IBL, Minneapolis, USA).

### Quantification of Abeta load

Plaque load was quantified in NT4X immunized 5XFAD mice and control groups. For each animal, 5–6 paraffin embedded sections, which were at least 80 μm afar from each other, were stained simultaneously with DAB as chromogen. For Thioflavin S fluorescent staining tissue sections were deparaffinized and rehydrated, washed twice in deionized water and then treated with 1% (w/v) ThioflavinS in aqueous solution and counterstained in a 1% (w/v) aqueous solution of 4´6-diamidin-2-phenylindol. The relative Aβ load was evaluated in the frontal cortex using an Olympus BX-51 microscope equipped with an Olympus DP-50 camera and the ImageJ software (NIH, USA). Representative pictures of 100× magnification were systematically captured. Using ImageJ the pictures were binarized to 8-bit black and white pictures and a fixed intensity threshold was applied defining the DAB staining. Measurements were performed for a percentage area covered by DAB staining, as well as for the number of grains per mm^2^ and the average size of the grains.

### Abeta ELISA

Brains were homogenized in 8-fold amount of TBS Lysis Buffer (50 mM Tris, pH 8.0 supplemented with 1 tablet/10 ml of Complete Protease Inhibitor cocktail (Roche)) and homogenates were spun down for 20 min at 17.000× g, 4 °C in a stratos Biofuge (Thermo Fisher). Supernatant (termed ‘TBS fraction’) was separated. The pellet was resuspended in 1 ml TBS lysis buffer and spun down again. Supernatant was discarded and the pellet dissolved in SDS lysis buffer (2% SDS supplemented with 1 tablet/10 ml Cømplete Protease inhibitor cocktail) using a branson sonifier 150 (Hellmann) at power ′2′ with 10 single pulses. The lysate was spun down again as described above and the supernatant (termed ‘SDS-Fraction’) was separated. Total protein concentrations of both fractions were determined using the RotiQuant Protein Assay (Roth) with BSA as a standard. ELISA of Aβ was carried out in 96-well high-binding microtiter plates as follows. For Aβ_1-40/42_, monoclonal antibody IC16^40^ raised against residues Aβ_1-15_ was used as a capture antibody (diluted in PBS pH 7.2) and incubated over night at 4 °C in a humid chamber. After removal of the capture antibody, brain lysate samples (100-fold diluted for Aβ_1-40_; 1000-fold diluted for Aβ_1-42_) were added. Antibodies specific for the C-terminal Aβ_x-40_ or Aβx-42 epitope coupled to horseradish peroxidase diluted in PBS (pH 7.2, supplemented with 0.05% Tween 20, 1% BSA) were used as secondary antibodies and again incubated over night at 4 °C in a humid chamber. After three times washing with PBS (pH 7.2, supplemented with 0.05% Tween-20, 1% BSA), 50 μl of TMB ELISA peroxidase substrate (Interchim) was added and incubated for 1–10 min at RT in darkness. The reaction was stopped with 50 μl 2 M H_2_SO_4_ and absorbance was measured in a Paradigm microplate reader (Beckman Coulter) at 450 nm. For generation of standard curves, synthetic Aβ_1-40_ and Aβ_1-42_ peptides (JPT Peptide Technologies) freshly dissolved in DMSO at 10 μg/mL were used. Aβ_pE3-42_ ELISA was performed using the Amyloid-beta (3pE-42) ELISA kit (JBL) according to the manufacturer´s instructions.

### Intracerebroventricular injection of soluble Aβ

12-week-old male C57BL/6J mice (Janvier, Le Genest-St-Isle, France) were intracerebroventricularly (icv) injected under anesthetization. Freshly prepared Aβ_4-42_ peptides [50 pmol in 1 μL; 0.1 M phosphate-buffered saline (pH 7.4)] or freshly prepared 50 pmol Aβ_4-42_ followed 10 min by injecting the NT4X antibody or its Fab fragment [0.1 M phosphate-buffered saline (pH 7.4)] was injected into the right ventricle, with stereotaxic coordinates from the bregma (AP –0.22, L –1.0 and D 2.5 in mm). Vehicle (0.1 M phosphate-buffered saline) or vehicle in combination with 1 or 10 pmol NT4X was injected into the right ventricle as a control. icv injections were made using a 10-μl Hamilton microsyringe fitted with a 26-gauge needle. Four days following icv infusion of Aβ peptides, working memory was assessed using the Y-maze test.

### Working memory by the Y-maze task

Immediate spatial working memory performance in ICV injected 12-week-old male C57BL/6J wild-type mice was assessed by recording spontaneous alternation behavior in a Y-maze as described previously^13^,^41^,^42^.

### Statistical analysis

Differences between groups were tested with one-way analysis of variance (ANOVA) followed by Bonferroni multiple comparisons, ANOVA followed by Dunnett’s multiple comparisons test or two-way repeated measures ANOVA as indicated. All data are given as means ± standard error of the mean (SEM) or standard deviation (SD) as indicated. Significance levels are given as follows: ***p < 0.001; **p < 0.01; *p < 0.05. All statistics were calculated using STATISTICA version 10.0 for Windows (StatSoft, Tulsa, OK, USA) and GraphPad Prism version 5.04 for Windows (GraphPad Software, San Diego, CA, USA).

### Study approval

Animal experiments were approved by the local animal protection authorities (Niedersächisches landesamt für Verbraucherschutz und Lebensmittelsicherheit) under the approval number 14/1450. The experiments were conducted in accordance with the approved protocols.

## Additional Information

**How to cite this article**: Antonios, G. *et al.* Alzheimer therapy with an antibody against N-terminal Abeta 4-X and pyroglutamate Abeta 3-X. *Sci. Rep.* **5**, 17338; doi: 10.1038/srep17338 (2015).

## Figures and Tables

**Table 1:** ELISA of brain lysates after therapeutic treatment using the antibodies NT4X, 1–57 and of PBS injected controls

	Aβ_1-40_[pg/mg]	Aβ_1-42_[pg/mg]	Aβ_pE3-42_[pg/mg]
PBS	28042 ± 9465	51604 ± 20483	119.1 ± 65.03
NT4X	27140 ± 15800	41314 ± 15490	89.30 ± 45.01
1–57	35406 ± 4568	61376 ± 13224	104.3 ± 38.32

One-way analysis of variance (ANOVA) followed by Dunnett’s multiple comparison test against the PBS control group; n = 7–8; data presented as mean ± SD.
